# Robust mosaicking of maize fields from aerial imagery

**DOI:** 10.1002/aps3.11387

**Published:** 2020-09-10

**Authors:** Rumana Aktar, Dewi Endah Kharismawati, Kannappan Palaniappan, Hadi Aliakbarpour, Filiz Bunyak, Ann E. Stapleton, Toni Kazic

**Affiliations:** ^1^ Department of Electrical Engineering and Computer Science University of Missouri Columbia Missouri USA; ^2^ Interdisciplinary Plant Group and MU Plant Science Foundry University of Missouri Columbia Missouri USA; ^3^ Missouri Maize Center University of Missouri Columbia Missouri USA; ^4^ Department of Biology and Marine Biology University of North Carolina Wilmington North Carolina USA

**Keywords:** aerial imagery, crop field imagery, maize, mosaicking, small unmanned aerial system (sUAS), video summarization

## Abstract

**Premise:**

Aerial imagery from small unmanned aerial vehicle systems is a promising approach for high‐throughput phenotyping and precision agriculture. A key requirement for both applications is to create a field‐scale mosaic of the aerial imagery sequence so that the same features are in registration, a very challenging problem for crop imagery.

**Methods:**

We have developed an improved mosaicking pipeline, Video Mosaicking and summariZation (VMZ), which uses a novel two‐dimensional mosaicking algorithm that minimizes errors in estimating the transformations between successive frames during registration. The VMZ pipeline uses only the imagery, rather than relying on vehicle telemetry, ground control points, or global positioning system data, to estimate the frame‐to‐frame homographies. It exploits the spatiotemporal ordering of the image frames to reduce the computational complexity of finding corresponding features between frames using feature descriptors. We compared the performance of VMZ to a standard two‐dimensional mosaicking algorithm (AutoStitch) by mosaicking imagery of two maize (*Zea mays*) research nurseries freely flown with a variety of trajectories.

**Results:**

The VMZ pipeline produces superior mosaics faster. Using the speeded up robust features (SURF) descriptor, VMZ produces the highest‐quality mosaics.

**Discussion:**

Our results demonstrate the value of VMZ for the future automated extraction of plant phenotypes and dynamic scouting for crop management.

Current technologies generate massive amounts of video data that could provide abundant spatiotemporal information for plant phenotyping and crop management. Like other robotic technologies, small unmanned aerial systems (sUAS), or drones, produce new types of high‐throughput data that are very difficult to generate using manual scouting and sampling (Furbank and Tester, 2011; White et al., 2012; Busemeyer et al., 2013; White and Corley, 2013; Andrade‐Sanchez et al., 2014; Müller‐Linow et al., 2015; Shi et al., 2016). The advantages of sUAS over ground‐based robots and structured fields include the minimal damage to plants, rapid data collection, low capital costs, high portability among field sites, and no soil compaction (Busemeyer et al., 2013; Andrade‐Sanchez et al., 2014; Vadez et al., 2014; Liebisch et al., 2015; Blue River Technology [http://www.bluerivert.com/]; LemnaTec GmbH [http://www.lemnatec.com/]; Maricopa Agricultural Center [http://cals‐mac.arizona.edu/precision‐agriculture/]). Recent research applying drone technologies to phenotyping crops (Huang et al., 2013; López‐Granados et al., 2016; Gnädinger and Schmidhalter, 2017; Nasir and Tharani, 2017) builds on the extensive experience of the remote‐sensing community (Rouse et al., 1974; Li et al., 2014; Liebisch et al., 2015; Minervini et al., 2015). Several diverse crops, including tomatoes (*Solanum lycopersicum* L.), wheat (*Triticum aestivum* L.), and maize (*Zea mays* L.), have been phenotyped for traits ranging from plant height to stress responses to vegetative parameters measured at the canopy level (Condorelli et al., 2018; Enciso et al., 2019; Gracia‐Romero et al., 2019; Johansen et al., 2019; Wang et al., 2019). As described by Shi et al. (2016), the most common flight trajectory is a tight serpentine over the field with the camera in nadir view. The vehicle is paused at precomputed imaging waypoints chosen to ensure a high overlap between successive pairs of images, and still images of the canopy are collected. The number of waypoints and the image resolution are determined by the vehicle’s altitude and the camera’s resolution. Especially for large fields, feasible flight times require higher altitudes and faster speeds, lowering the resolution of the imagery and increasing the likelihood of pixel blurring. Moreover, a nadir camera view ablates the morphological structures that lie beneath the canopy, which are visible in an oblique view.

The ultimate goal of quantitating phenotypes with aerial imagery begins with faster, more accurate methods for registering the flight imagery into a single mosaic that preserves the relative spatial position of each plant. Image registration, the first step in mosaicking, aligns two images of a scene taken from different viewpoints by identifying the same objects in each image, despite differences in their perspective, apparent position, and scaling. Mosaicking reconciles multiple views of the same field and the plants within it into a unified whole that can be compared with similar mosaics from imagery taken throughout the growing season. It can also be used to summarize a long video, enabling rapid searching and filtering without manual viewing (Viguier et al., 2015). Computer vision research has long focused on image registration and mosaicking (Brown, 1992; Zitova and Flusser, 2003); for example, image registration is widely applied to remote sensing, biomedicine, computer vision, and cartography (Pohl and Van Genderen, 1998; Chen et al., 2003; Zitova and Flusser, 2003; Bentoutou et al., 2005; Li et al., 2009; Aktar et al., 2016, 2018b; Teters et al., 2018; Ufuktepe et al., 2020). Each video frame has its own local coordinate system, which must be optimally transformed into the mosaic’s global coordinate system using a sequence of *homography* matrices that map each local system to that of the adjacent image, and thence to the global coordinate system.

The two main approaches to computing homographies involve detecting distinctive, corresponding features (*key points*) between images using highly localized statistical descriptors (Schonberger and Frahm, 2016). The first approach computes image‐to‐image homography between successive views in two‐dimensional (2D) space, assuming the field of interest is adequately planar, and the homographies are multiplied together to produce the mosaic. Minor errors in determining each matrix rapidly accumulate as spatial and registration errors during mosaicking, and additional drift correction using non‐image metadata has been required to rapidly produce accurate mosaics (Molina and Zhu, 2014). These external metadata include georegistration or external or vehicle global positioning system data; camera and vehicle position and orientation from the vehicle’s inertial measurement unit and gimbal orientation sensors; or ground control points (i.e., fiducial markers placed on the ground to provide relative registration) (Zhu et al., 2005; Lin and Medioni, 2007). Tightly constraining trajectories and camera poses further simplify the computations by permitting the precomputation of the homographies. Downsampling the imagery produces smaller data sets that can be registered more quickly. Importantly, current phenotyping studies use either Pix4DMapper (Pix4D, Prilly, Switzerland) or AgiSoft (AgiSoft, St. Petersburg, Russia), both of which rely heavily on these strategies to produce canopy mosaics (Shi et al., 2016; Condorelli et al., 2018; Enciso et al., 2019; Gracia‐Romero et al., 2019; Johansen et al., 2019; Wang et al., 2019).

Unfortunately, this approach imposes substantial technical and resource requirements. The high‐performance custom vehicles, sensors, and data acquisition systems that produce the metadata are expensive to purchase and use. Downsampling reduces image resolution, jeopardizing the early, sensitive detection of phenotypes and management problems. The analysis of large fields using this technique requires more and longer missions with more burdensome flight planning, field preparation, and data integration. Most importantly, fixed trajectories and nadir camera poses preclude the use of orbital trajectories and oblique poses that can produce other kinds of information, e.g., for 3D reconstruction of morphological phenotypes (Seetharaman et al., 2019; Aliakbarpour et al., 2020), and they cannot change to higher‐resolution imaging when anomalies are detected. Rapidly surveying large fields with variable trajectories and resolutions would permit automated scouting, reducing flight time and pilot fatigue.

The second approach to computing homographies permits unconstrained trajectories and freedom from metadata, but sharply increases computational effort. Structure from Motion (SfM) uses only imagery to solve for the 3D scene and the camera spaces, globally optimizing both to more accurately recover camera poses and a sparse 3D point cloud (Agarwal et al., 2011; Gao et al., 2018). Thus, this approach can be applied to landscapes without ground control points imaged with inexpensive consumer‐grade equipment that lacks telemetry, flown without constraining the trajectory to a set of predetermined imaging waypoints (“freely flown”). The computational complexity of SfM grows exponentially with the number of frames in a data set (Agarwal et al., 2011; Wu, 2013), making it prohibitively expensive for data with hundreds of frames.

Computing homographies for even small crop fields can be very challenging, and both approaches have additional difficulties when compared with mosaicking man‐made built environments (AliAkbarpour et al., 2015, 2017). First, built environments have many unique, sharp‐edged features that remain static during image capture and are minimally repeated over the landscape of interest, enabling the use of edge‐detection algorithms. In contrast, crop fields are composed of thousands of non‐sharply edged plant features that are very similar to each other. Plants repeat over the entire field, so plants in one frame look very much like those in adjacent frames. These repetitive features reduce the number of corresponding key points the algorithm correctly recognizes, misestimating the homographies and degrading registration. Although biological differences among the plants exist and occlusion during imaging occurs, these are not sufficient to give every plant visually or statistically distinct features. Second, plant features can change during the capture of a single video frame. The intermittent, irregular, and nonsmooth movements of plants due to local air currents and propeller downdraft complicate feature detection and matching. Third, unplanned changes in vehicle trajectory due to local wind currents or the miscalculation of the field of view can suddenly change camera position before automated or manual correction can occur. These complications can produce significant errors in estimating the homographies that then accumulate as the entire mosaic is computed. A few random frames from our aerial videos of a maize nursery illustrate some of these problems (Fig. 1).

**FIGURE 1:**
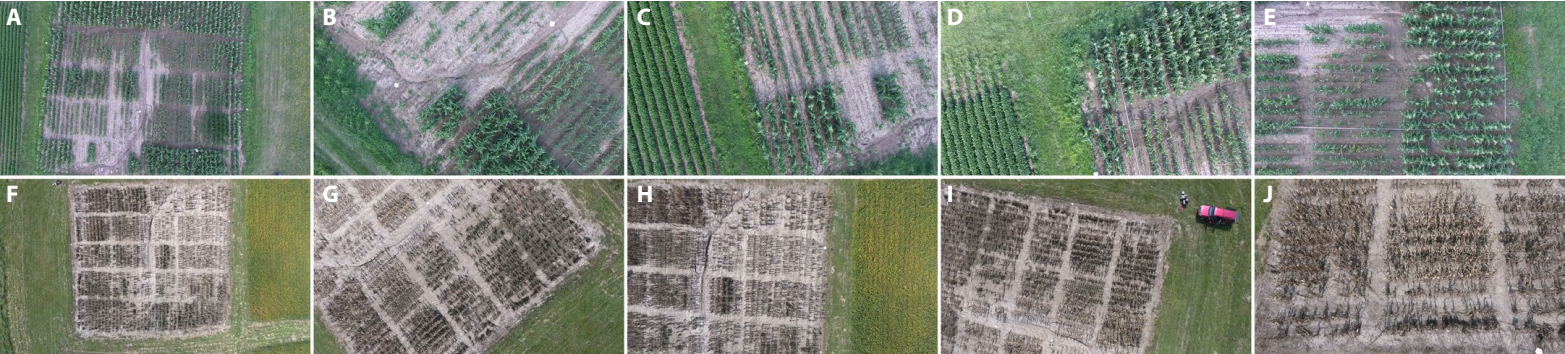
Representative images from two aerial video sequences of a maize field taken at different times during the growing season, each with 670 frames. (A–E) DJI_0026.mov; (F–J) DJI_0003.mov. (A) Frame_0001, (B) Frame_0355, (C) Frame_0443, (D) Frame_0537, (E) Frame_0631, (F) Frame_0001, (G) Frame_0167, (H) Frame_0240, (I) Frame_0308, (J) Frame_0664. The viewpoint changes as the aerial vehicle and camera position shift during flight. For both sequences, the first image is a nadir view of the field.

We have developed a novel mosaicking procedure that produces accurate mosaics of agricultural imagery taken during freely flown flights by an inexpensive, easy‐to‐fly sUAS with an RGB camera. This Video Mosaicking and summariZation (VMZ) pipeline performs 2D registration to produce a mosaic representing the whole scene in a rapid and robust manner, without any metadata or drift correction. The VMZ pipeline accelerates the computations and minimizes frame‐to‐frame drift in three ways: (1) by exploiting the temporal ordering of the image frames to help find corresponding features; (2) by generating a set of mini‐mosaics from the video sequence and then referencing these to the base frame using a novel algorithm; and (3) by choosing the strongest key points detected with feature correspondence algorithms that are less confused by repetitive structures. Using maize research nurseries imaged in two different field seasons, we compared the performance of different feature descriptors for these videos. The VMZ pipeline with the speeded up robust features (SURF) descriptor much more efficiently and accurately produced mosaics than a well‐known stitching algorithm, AutoStitch (Brown and Lowe, 2007; MATLAB, 2019).

## MATERIALS AND METHODS

### Materials

A field of maize inbred lines, disease lesion mimic mutants, and elite corn was imaged twice during the 2016 growing season, once at maturity (DJI_0026.mov) and once after senescence (DJI_0003.mov). This field was planted at several different densities. In 2019, we imaged a field planted with the same inbred and elite genotypes, and closely related mutant genotypes, with rows planted at two different densities (DJI_0174.mov). In all fields, the rows were 20 ft (6.1 m) long. Each block of parallel rows stretching across the field was a range, and the ranges were separated by 4 ft (1.2 m) alleys. Together, the rows and alleys form a visual checkerboard; the rows intersect the alleys at right angles.

### Imaging using the sUAS

All imaging was performed near solar noon using a DJI Phantom 4 (Da‐Jiang Innovations, Shenzhen, China) photography drone. No alterations to the vehicle or its camera were made, and all flights were flown manually. The trajectory for DJI_0026.mov was a fairly high‐altitude, two‐pass, right‐angled serpentine connected by an orbit; the camera was mostly held in a nadir pose. The trajectory for DJI_0003.mov was mostly a high‐altitude orbital trajectory with nadir and oblique camera views (Appendices S1, S2). DJI_0174.mov was taken with the camera in nadir view using a straight out‐and‐back trajectory at a low, relatively constant altitude (Appendix S3). Some lateral motion of the vehicle relative to the field occurred during the course of the flight. All trajectories involved marked changes in altitude, the direction of the vehicle relative to the field, and the camera pose.

The 1.0‐megapixel videos were recorded at 24 frames/s in 24‐bit color. The video files were written on Secure Digital High Capacity (SDHC) cards carried on the sUAS and manually copied onto the computer used to generate the mosaics. This machine had an Intel Core i7 processor running at a clock speed of 2.2 GHz, and 32 GB of memory.

### Video preprocessing

The correction of the Phantom 4’s lens distortion was performed using the MATLAB calibration toolbox developed by Bouguet (Bouguet and Perona, 1998; Bouguet, 2015). The procedure returns camera intrinsic, extrinsic, and distortion parameters that are then applied to the raw frames prior to mosaicking. A calibration checkerboard of 12 × 9‐square checks (each check is 60 × 60 mm; Calib.io, Svendborg, Denmark) was imaged from different angles of view and camera positions relative to nadir. Errors in image selection, the manual definition of the checkerboard’s corners, or automated corner extraction produce large pixel errors. These problems must be corrected prior to the iterative optimization of the initial estimates. We refined ours thrice to get a minimum average pixel error.

The computation of feature points and matching is often a huge bottleneck in terms of time complexity. The original video frame sizes were 3840 × 2160 pixels (DJI_0003.mov and DJI_0026.mov) and 4096 × 2160 pixels (DJI_0174.mov). DJI_0003.mov and DJI_0026.mov were downsampled to 1920 × 1080 pixels, while DJI_0174.mov was corrected for lens distortion prior to downsampling to 2018 × 1047 pixels. No other preprocessing was performed. These corrected downsampled frames were directly used for shot detection and mosaic generation.

### Shot detection

**FIGURE 2:**
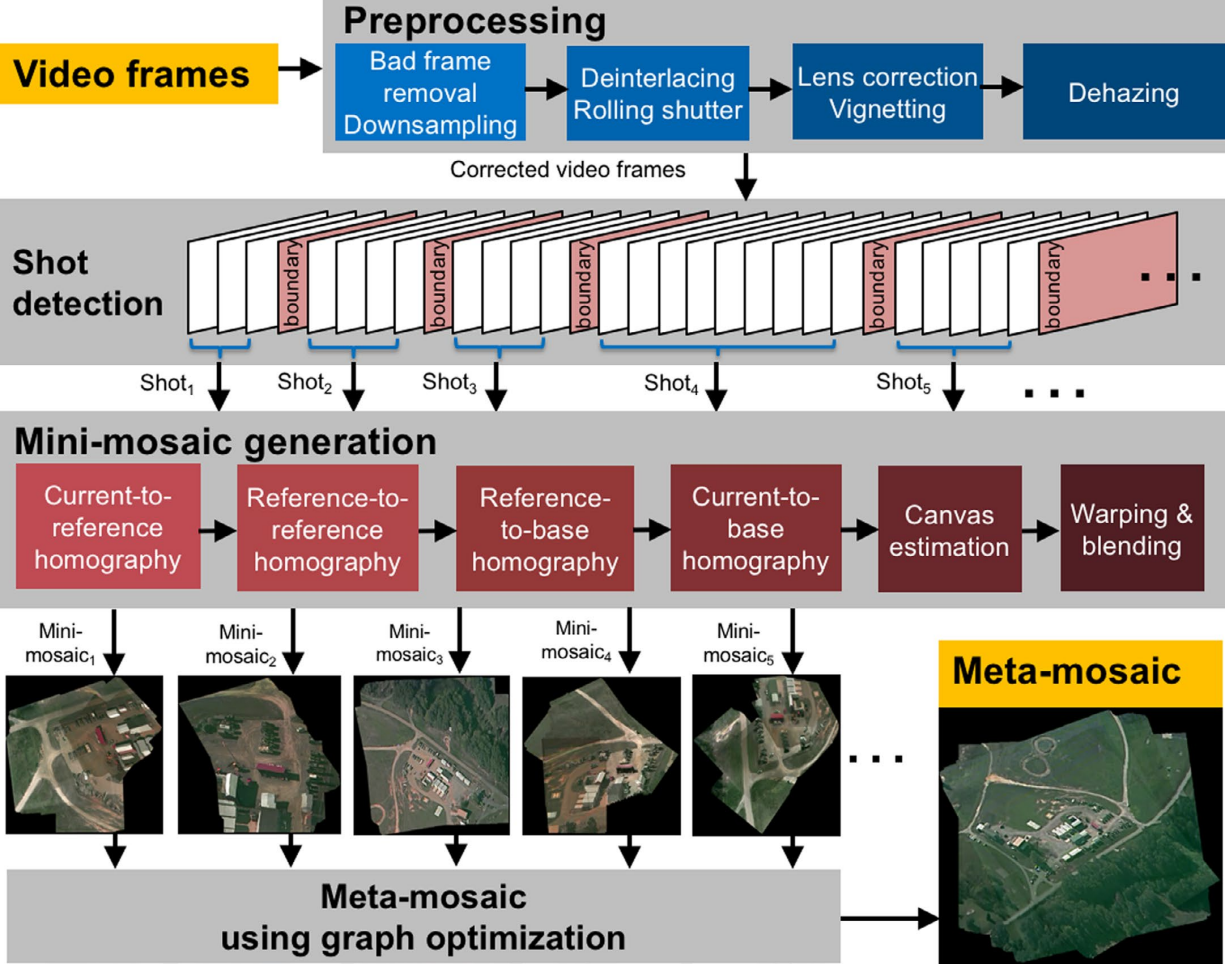
The VMZ pipeline. The main steps in the pipeline are: preprocessing, video segmentation based on shot detection, mini‐mosaic generation, and meta‐mosaicking using a graph optimization‐based method.

The first step in our mosaicking algorithm is to segment the image sequence into a few meaningful spatially and temporally overlapped segments based on scene changes and camera motion. A meaningful segment is called a shot, and this method is called shot detection (second row in Fig. 2). The input is the cumulative histogram difference in graph‐cut energy (Viguier et al., 2015) between consecutive frames of the whole video. These differences mainly form two clusters of frames. The algorithmic details are provided in Appendix 1.

### Feature detection for frame‐to‐frame registration

The next step is to find distinctive features in one image and identify their corresponding features in a subsequent image. Features can be corners, edges, contours, textures, intersection points, etc., and can be described mathematically by many different strategies. Here, we briefly describe the four standard feature descriptors we tested; fuller descriptions are given in Appendix 1.

A 2D color structure tensor (ST) (Hafiane et al., 2008) is a semi‐local descriptor that finds large, distinctive properties of an image, such as edges and corners, over a microblock of pixels. Microblocks in one image are compared to those in another using a search window, returning the positions of the similar blocks (Hafiane et al., 2008). Scale‐invariant feature transform (SIFT), SURF, and affine SIFT (ASIFT) are local descriptors that employ different strategies and statistics for feature matching and are widely used (Lowe, 2004; Bay et al., 2006; Morel and Yu, 2009). The SIFT descriptor is invariant to scaling, orientation, and illumination changes, and partially invariant to affine distortions, whereas SURF is invariant to changes in rotation, scale, and illumination, and to small changes in camera viewpoint. The ASIFT descriptor is a more robust version of SIFT that is invariant to scaling, rotations, and translations and can use the camera orientation parameters to find the transition tilt as the camera changes position.

The VMZ pipeline implements an adaptive feature descriptor (VMZ‐Adaptive) that combines features with different characteristics. Fast features such as ST and SURF are used for the efficient matching of images with large overlap. When the overlap between frames is not sufficient, VMZ automatically switches to a more robust descriptor, ASIFT. This adaptive nature helps VMZ obtain good matches by compromising time efficiency a bit.

### Estimation of the homographies of the transformation models

Once the features that correspond in two images are identified, we use homography to geometrically map one frame to another. Given a landscape that is approximately planar, homography maps four points in one image into the coordinate system of another. The second coordinate system is described with reference to that of the first. The mathematical details of this mapping are provided in Appendix 1.

### Inlier selection using random sample consensus

Although the prior steps produce a large number of matched, transformed features, at least some of these matches are poor or artifactual. Random sample consensus (RANSAC) is used to establish a robust matching between images by removing putative matches that are outliers in an estimated distribution (Fischler and Bolles, 1987). Random subsets of the matches are used to iteratively fit a linear model; those points that fit the proposed model are the retained “inliers.”

### Pixel filling

In this method, any pixel in the canvas comes from a single frame. Pixels that are not present in the canvas at time *t* − 1 but are found in the warped frame at time *t* are added to the mosaic.

### Evaluation of mosaic quality and algorithm performance

We evaluated the performance of VMZ in three ways: inspection, quantitative performance, and comparison to AutoStitch (Brown and Lowe, 2007; MATLAB, 2019). Quantitative comparisons of mosaic quality used the structural similarity indices *SSIM_f_* and *SSIM_p_* and scene integrity (Bolaños and Edmeades, 1996; Yang et al., 2015) (for computational details, see Appendix 1); *SSIM_f_* quantifies how well a warped frame at time *t* is aligned or structurally similar to an overlapping region of the initial mosaic computed at *t* = 1, while *SSIM_p_* measures how structurally similar a warped frame at time *t* is to the mosaic obtained from frames 1 to *t* − 1. Scene integrity is defined as the ratio of images in the mosaic that match those in the input scene; scores range from 0 (complete failure) to 1 (complete success). The run time performance in Table 1 was benchmarked as a function of image sequence, feature descriptor, and the average number of features in each frame. AutoStitch is a standard 2D registration algorithm incorporated into several commercial products. The MATLAB implementation uses the SURF descriptor, but unlike VMZ‐SURF it uses all image features to establish image matching (Brown and Lowe, 2007; MATLAB, 2019).

**TABLE 1:** Quantitative comparison of the different methods for mosaicking the three maize videos. The best results for run time and the structural similarity index measure (SSIM) statistics are in bold.

Data set^a^	Feature	Features/frame^b^	No. of images	Scene integrity	Time (s)	s/Frame	*SSIM*_f_	*SSIM*_p_
DJI_0026	AutoStitch	13,560	670	1	2195	3.28	—	—
	VMZ‐Adaptive	857	670	1	14,726	21.98	0.9734	0.9803
	VMZ‐ASIFT	35,780	670	1	38,964	58.16	**0.9911**	0.9985
	VMZ‐SURF	5000	670	1	**1331**	**1.99**	0.9899	**0.9990**
DJI_0003	AutoStitch	13,706	670	1	1587	3.86	—	—
	VMZ‐Adaptive	857	670	1	15,316	22.86	0.9413	0.9883
	VMZ‐ASIFT	36,344	670	1	51,673	77.12	0.9401	0.9885
	VMZ‐SURF	5000	670	1	**1540**	**2.29**	**0.9491**	**0.9984**
DJI_0174	AutoStitch	12,890	151	1	485	3.21	—	—
	VMZ‐Adaptive	807	151	1	3436	22.75	0.9802	0.9812
	VMZ‐ASIFT	34,040	151	1	9097	60.25	0.9956	0.9978
	VMZ‐SURF	5000	151	1	**352**	**2.33**	**0.9957**	**0.9978**

Note

*SSIM*_f_ = how well a warped frame at time *t* is aligned or structurally similar to an overlapping region of the initial mosaic computed at *t* = 1; *SSIM*_p_ = how structurally similar a warped frame at time *t* is to the mosaic obtained from frames 1 to *t* − 1.

^a^ Mosaics for the sequences DJI_0003 and DJI_0174 are presented in Appendixes S1 and S3.

^b^ The number of structure tensor (ST) features for the adaptive feature descriptor (VMZ‐Adaptive), which switches between the ST, speeded up robust features (SURF), and affine scale‐invariant feature transform (ASIFT).

## RESULTS

### Overview of the VMZ pipeline

**FIGURE 3:**
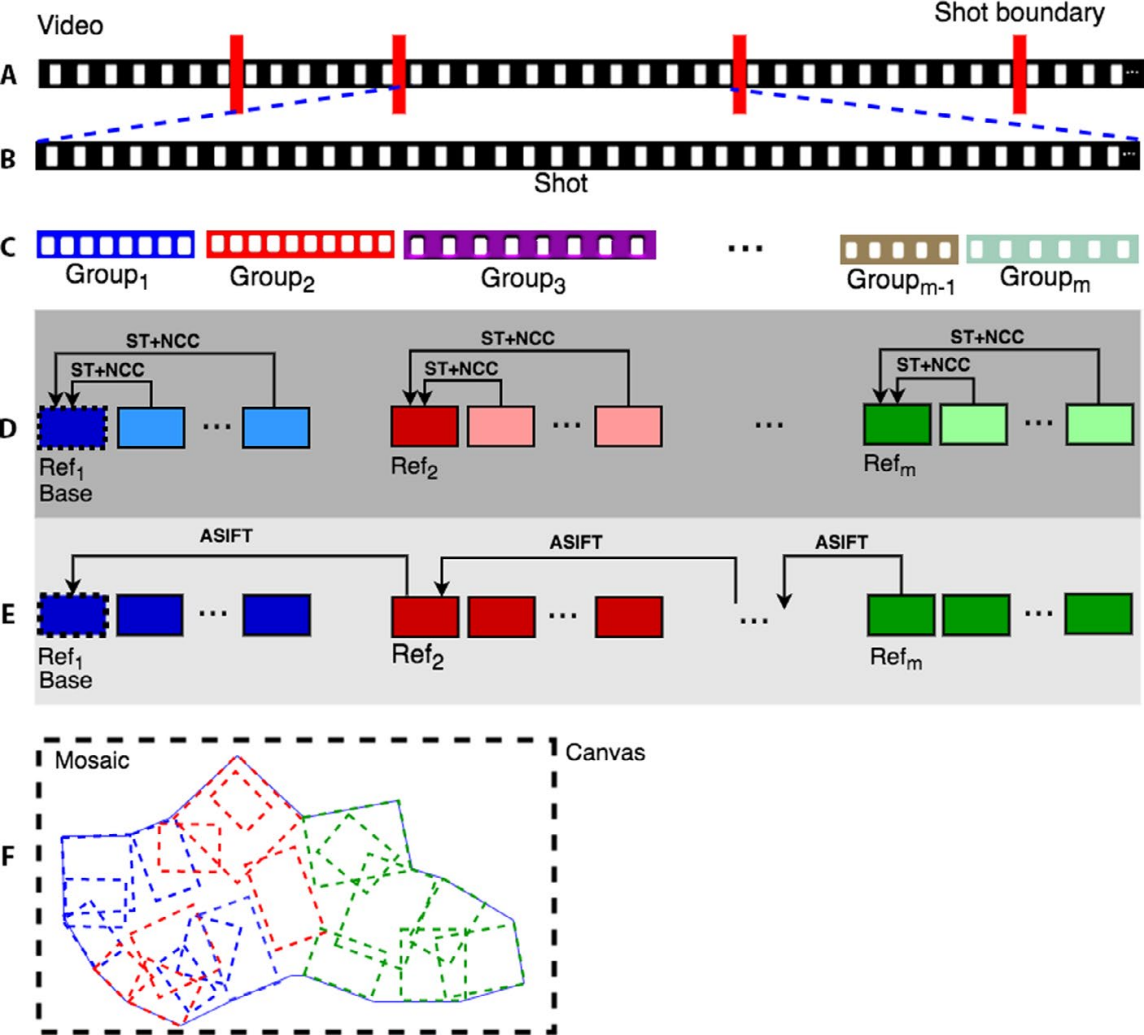
The VMZ transformation model. The VMZ transformation model explains how images are registered after shot boundaries are found. The red bars in row A represent a shot boundary. Once a shot is detected (row B), its frames are divided into a few groups using frame motion information and the number of frames in a group (row C). Each group is represented as a single color (*Group*_1_ is in blue in rows C and D), with the reference frame (first frame of the group) in the darkest shade (dark blue for *Group*_1_). Within a group, each frame is directly mapped to the reference by homography, as shown by the arrows (current‐to‐reference mapping; row D). Next, each reference frame is homographically mapped to the previous reference in order to obtain a global coordinate system for all frames (reference‐to‐reference mapping; row E). The mosaic is generated in the coordinates of the base or first frame (row F). Base, first frame of a shot; Ref, reference frame.

We present a complete system, VMZ, that accepts a sequence of video frames as input and produces one or more mosaics from the sequence. The block diagram of the VMZ pipeline is presented in Fig. 2. The video preprocessing and shot detection steps in VMZ are described in the Methods. Below, we describe our mini‐mosaicking algorithm. The algorithm’s pseudocode is presented in Appendix 1 (Algorithm 1: Mini‐mosaic generation) and is also shown pictorially in Fig. 3.

### Steps in mini‐mosaic construction

Algorithm 1 (Appendix 1) includes three main steps: homography calculation, canvas estimation, and finally warping and blending. A very preliminary version of the mini‐mosaicking algorithm was presented by Aktar et al. (2018a, b).

- **Homography estimation:** The homography module estimates four different homography matrices for warping all the frames together. Within a group, it directly maps each frame to the first frame of the group, which is referred to as the *reference* frame. This mapping relation is denoted as current‐to‐reference homography (*H_CR*) (line 7). If the number of frames in the current group is greater than 40 or the displacement between the current frame (${\mathrm{Frame}}_{i}$) with respect to the group’s reference frame is greater than the threshold (one‐third of frame height or width), then a new group is formed (Algorithm 1, lines 8–13). Next, the reference‐to‐reference homography (*H_RR*), which aligns any group’s reference frame to that of the preceeding group, is calculated directly (line 9). The reference‐to‐base homography (*H_RB*), which aligns a reference to the first frame of a shot, is estimated using the *H_RR* between the current frame and the reference frame, and the *H_RB* between the prior frame and its prior frame by Eqn. 7 (Algorithm 1, line 11). Finally, the current‐to‐base homograpy, which aligns the current frame to the reference frame of the group, is estimated from *H_CR* and *H_RB* by Eqn. 8 (Algorithm 1, line 15).
- **Canvas estimation:** Canvas size estimation is straightforward. We transform the four corners of each frame with the current‐to‐base homography (*H_CB*) to get the bounding box information of the image in mosaic coordinates. From the bounding box information, we need the four extremal points: the leftmost lower and upper corners, and the rightmost lower and upper corners (Algorithm 1, lines 18–19). These four points define the canvas size.
- **Warping and blending:** We warp each frame by inverting its corresponding current‐to‐base homography (*H_CB*) (Algorithm 1, line 21). Next, we extract a region of interest (ROI) from the current mini‐mosaic using the corners of the current frame, *i* (Algorithm 1, line 22). Once we have the ROI, we blend using the pixel‐filling method described above. Finally, we reassign or update the mini‐mosaic canvas (Algorithm 1, line 24).

### Global evaluation of feature descriptors

Quantitative comparisons of VMZ’s feature descriptors and AutoStitch for the maize imagery are shown in Table 1. AutoStitch uses all the image features to establish sequential matching, identifying more features than VMZ‐Adaptive and VMZ‐SURF at the cost of consistently slower run times than VMZ using any feature descriptor. In VMZ‐Adaptive, the ST descriptor finds the edges and corners in an image, grouping them into feature blocks (around 800 per frame). When the percentage of matched features after RANSAC elimination (the percent inliers) falls below 40%, VMZ‐Adaptive switches from the ST to the SURF descriptor. Because it uses ASIFT only for reference frame matching, VMZ‐Adaptive is faster than ASIFT but slower than SURF. The ASIFT descriptor extracts the most points of all the descriptors, improving feature matching quality at the cost of long computational times. Finally, SURF extracts around 14,000 feature points but picks the strongest 5000 key points for matching and registration. As a result, its computational complexity and time is much lower than ASIFT.

We quantitated the mosaic quality for VMZ with each descriptor using *SSIM_f_* and *SSIM_p_*, which require the generation of an intermediate mosaic at each individual frame or a known ground‐truth mosaic. Unlike VMZ, AutoStitch does not provide these additional data, so we could not compute these statistics for it. Usually *SSIM_f_* was highest for VMZ‐SURF, with VMZ‐ASIFT performing marginally better for DJI_0026.mov. For all three videos, *SSIM_p_* was highest for VMZ‐SURF. The scene integrity was the same for all algorithms. Judged by the mosaic‐quality scores and speed, SURF was the most useful method for extracting features and matches for these maize video sequences.

**FIGURE 4:**
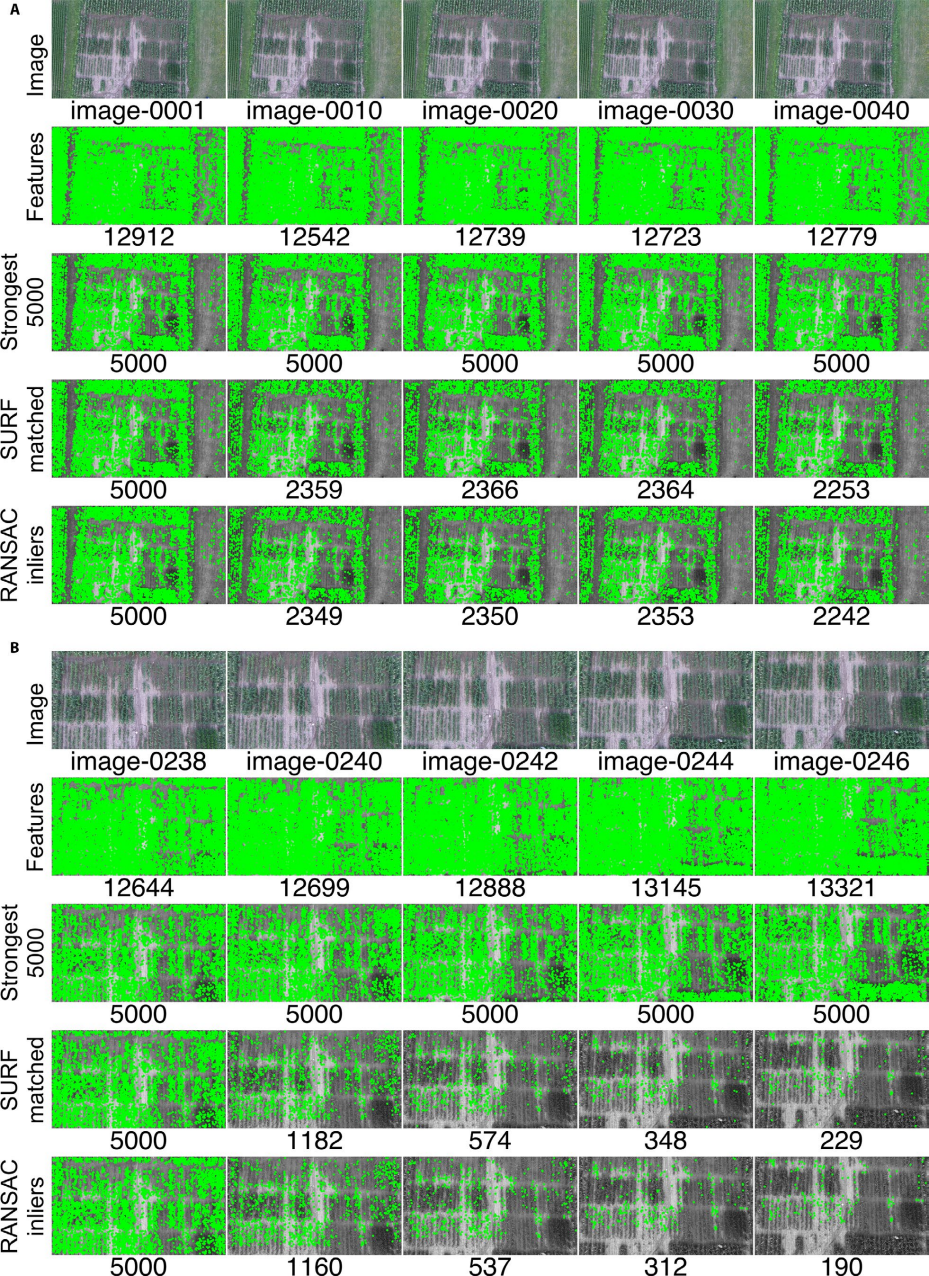
Changes in the SURF point distribution during VMZ processing in frames of DJI_0026.mov. (A) *Group*_1_, shots of a video flown with a slow trajectory. (B) *Group*_6_, shots flown with a fast trajectory. The shots were sampled at every 10th (A) and second (B) frame. The location of each feature is shown by a green dot. The number of features is shown at each step below the frames. Rows from top to bottom: the original image with its frame number below it (Image), all features extracted by SURF (Features), the strongest 5000 SURF features (Strongest 5000), features matched with the reference frame features by SURF matching (SURF matching), and finally, inliers after filtration by RANSAC (RANSAC inliers).

Examples of the features SURF finds after extraction, matching, and inlier selection for slow and fast platform trajectories are shown in Fig. 4A and 4B, respectively. The top row in each panel shows the raw frames. The second and third rows contrast the features extracted by SURF and show the strongest 5000 features. The fourth row shows the features matched by SURF, and the bottom row presents just the inliers found by RANSAC. The difference in trajectory speeds affects feature matching in VMZ, as shown in the SURF matched row; more than 2300 matches were found in the image groups captured at slower speeds, dropping to 229 matches for fast speeds.

We also examined how well each of VMZ’s descriptors function during the pipeline and how well SURF performed over the sequences DJI_0026.mov and DJI_0003.mov (Appendix S4). For both videos, ST extracted the fewest features and relied most heavily on the RANSAC inliers. The most features were extracted using SURF, which relied least on RANSAC; ASIFT fell in between.

### Local mosaic quality

**FIGURE 5:**
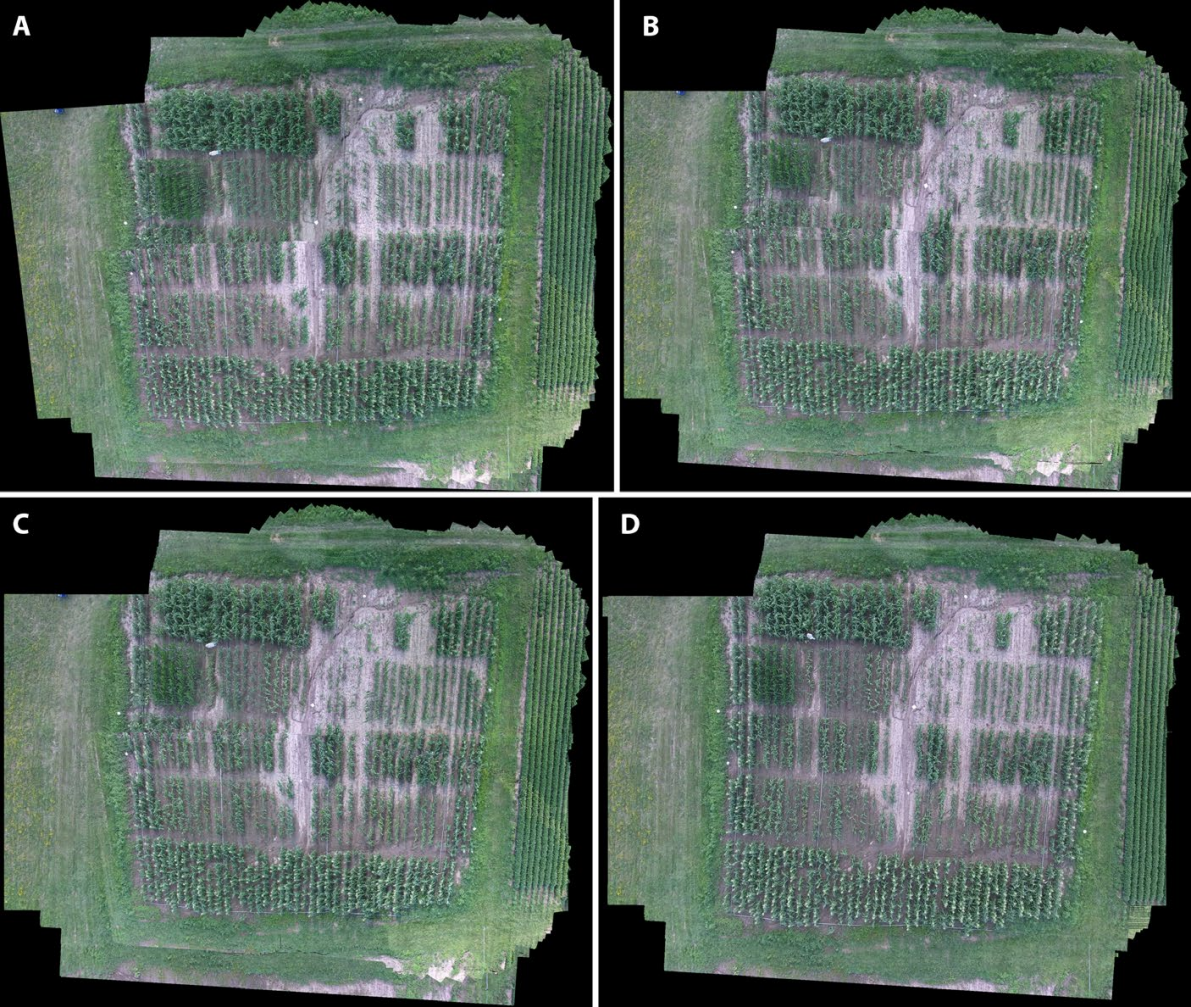
Mosaics of sequence DJI_0026.mov produced by AutoStitch (A) and VMZ’s three feature descriptors (B–D). (A) AutoStitch (3319 × 2654 pixels), (B) VMZ‐Adaptive (2005 × 1723 pixels), (C) VMZ‐ASIFT (2022 × 1737 pixels), (D) VMZ‐SURF (2006 × 1723 pixels). Sizes in parentheses are of the original high‐resolution mosaics; they are rescaled in the figure to make the field approximately the same size in each mosaic. At this scale, registration errors are visible (A–C) as a jagged left edge and foreshortening of the lower right corner of the field. Minor lens distortion is visible along the bottom of the mosaics.

**FIGURE 6:**
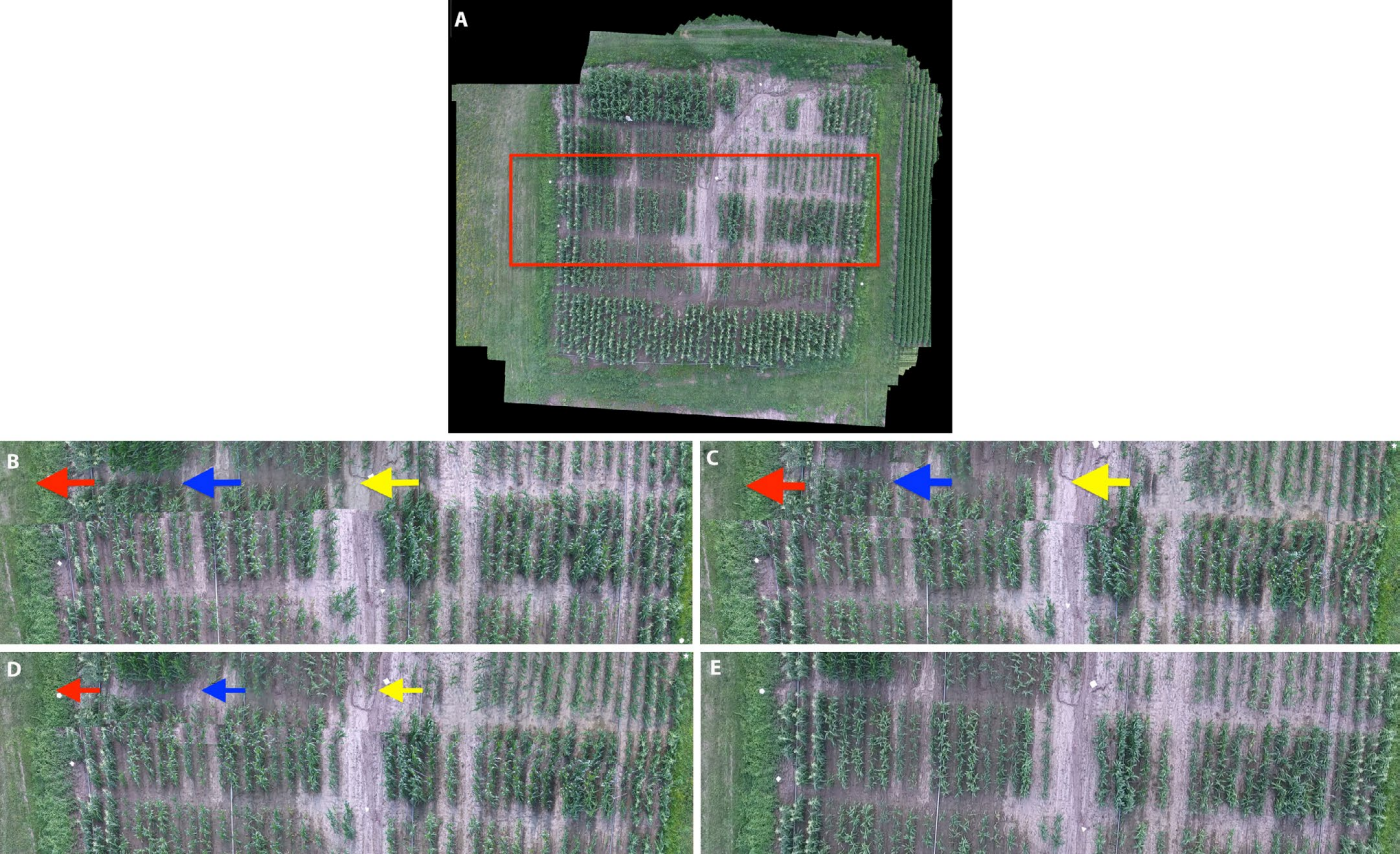
Examples of registration errors in the DJI_0026.mov mosaics. In (A), the red box marks a region from the VMZ‐SURF mosaic that is expanded in (B–E) for AutoStitch and VMZ’s three feature descriptors: (B) AutoStitch, (C) VMZ‐Adaptive, (D) VMZ‐ASIFT, (E) VMZ‐SURF. The approximate global registration error is seen as an offset along the segments of the three physically continuous irrigation pipes; these are marked with arrows in (B–D). These global errors are also visible as an artificial boundary in the center of the mosaics. Arrow color denotes the magnitude of the errors; red, worst; blue, moderate; yellow, least bad. Local registration errors are visible as the blurring of the plants’ leaves and tassels and the apparently curved rows on the right side of the field.

The global statistical descriptors provide a rough estimate of overall mosaic quality, but absent comparing the mosaics over their entire extent to a ground‐truth image, the best way to assess quality is to closely examine the mosaics for localized errors. For each video sequence, we compared the mosaics produced by AutoStitch, VMZ‐Adaptive, VMZ‐ASIFT, and VMZ‐SURF (see Figs. 5, 6; Appendices S1, S2, S3) illustrating three types of artifacts: distortion of field geometry, global and local object mismatches, and color anomalies. Considering all data sets and types of errors, VMZ‐SURF produces the best mosaics. More details on mosaic geometry and feature statistics (as illustrated in Appendices S1–S4) are provided in Appendix S5.

#### Distortion of field geometry

Distortions due to mosaicking errors are visible in Figs. 5 and 6. In Fig. 5A–C, the bottom half of the actually straight left edge of the field was shifted leftward due to an abrupt discontinuity in the mosaic. Similarly, rows in the bottom right corner are foreshortened. Both VMZ‐Adaptive and VMZ‐ASIFT recovered the field’s perimeters and internal geometry more accurately than AutoStitch, although VMZ‐Adaptive (Fig. 5B) had more trouble with the right edge of the field than VMZ‐ASIFT (Fig. 5C), which foreshortened the right side of the bottom edge to a greater extent. Both had difficulty with the row/alley checkerboard. None of the three approaches were aided by the placed fiducial marks (white shapes mounted on posts). In contrast, VMZ‐SURF consistently produced mosaics with the least geometric distortion for sequences DJI_0026 (Fig. 5D) and DJI_0003 (Appendix S1). In other experiments, we corrected some of this trapezoidal distortion by correcting the first frame for gimbal distortion prior to mosaicking the sequence (data not shown). The remaining distortion is probably due to the nonplanarity of this field, which is crowned near the middle of the bottom edge and slopes down toward the corners. This suggests that the VMZ descriptors are more sensitive to field nonplanarity than AutoStitch’s full set of SURF key points.

#### Global and local object mismatches

Figures 5 and 6 illustrate two scales of mismatches by the four algorithms. The first is a global mismatch in the central range of the field, visible as a line across the range that produces the jagged left edge discussed above. In Fig. 6, this central region is highlighted in the VMZ‐SURF mosaic (Fig. 6A) and cropped and expanded for all four mosaics (Fig. 6B–E). The mismatches are visible as sudden offsets in the three irrigation pipes running parallel to the rows, highlighted by the arrows. The offsets produced were greatest for AutoStitch, followed by VMZ‐Adaptive and least for VMZ‐ASIFT. In contrast, VMZ‐SURF (Fig. 6E) registered the central range almost perfectly.

Local mismatches are visible on the right side of Fig. 6B–E. These manifest as two artifacts: blurred plants and curved rows. Both VMZ‐Adaptive and VMZ‐ASIFT blur the plants more than AutoStitch and especially VMZ‐SURF, which renders the sharpest plants over the entire field. The VMZ‐Adaptive, VMZ‐ASIFT, and VMZ‐SURF approaches (Fig. 6C–E) combined several rows to their adjacent rows in the middle of the range toward the right edge of the field. The magnitude and number of “curved rows” was greatest for VMZ‐Adaptive, then VMZ‐ASIFT, then VMZ‐SURF. AutoStitch exhibited the fewest local mismatches (Fig. 6B).

#### Color anomalies

The VMZ pipeline adds new pixels from any frame that are not present in the growing mosaic. In contrast, AutoStitch blends overlying frames to produce a mosaic in the global canvas. The pixel‐blending algorithm for the VMZ pipeline is therefore simpler than that of AutoStitch, which makes errors in mosaicking readily apparent. These anomalies are visible in the lower right corner of the mosaics produced using AutoStitch, VMZ‐Adaptive, and VMZ‐ASIFT (Fig. 5A–C). The grass below the maize and soybean (*Glycine max* L.) fields, and stripes in the soybeans at the edge of the maize field, are presented as yellow instead of green. By contrast, VMZ‐SURF displays much less misregistration, although some is still visible at the bottom of the soybean field (Fig. 5D).

### Mosaic footprints

**FIGURE 7:**
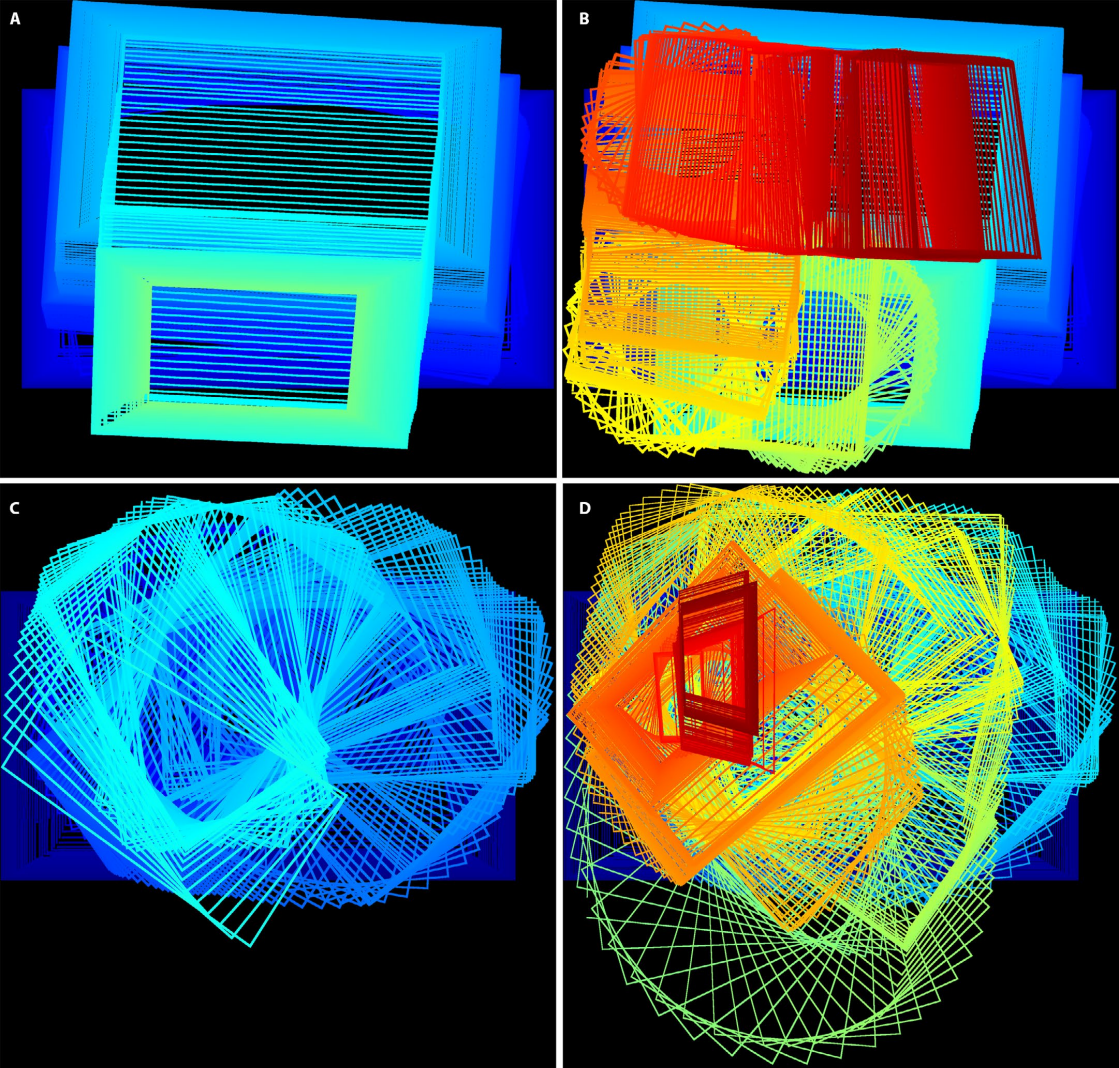
Footprints during mosaicking of DJI_0026.mov (A, B) and DJI_0003.mov (C, D). The first 330 (A) and all 670 frames (B) of DJI_0026.mov. The first 330 (C) and all 670 frames (D) of DJI_0003.mov. The color changes from blue to green to yellow to red as a function of time in the video sequences to increase visual clarity.

Finally, another way to assess the quality of the mosaicking is to determine the footprint, or transformed image boundary, of each frame placed in the final canvas. Because the vehicle’s trajectories are relatively smooth and continuous, each frame should stack fairly regularly in a good mosaic, allowing for affine transformations as shown in Fig. 7 for the VMZ‐SURF mosaics. The footprints for each video are divided into two sections, so that those from earlier in the video (Fig. 7A, C) are not obscured by the later frames (Fig. 7B, D), illustrating how well the transformed frames cover the final mosaic.

## DISCUSSION

Imagery of row crops, either in genetic nurseries or production fields, by definition contains large numbers of similar image features that repeat over the field. These confuse matching for registration and produce mosaicking errors, making the proper selection of the feature descriptor very important. The different VMZ descriptors and a standard mosaicking algorithm all exhibited characteristic registration errors for both the field and the individual plants. By selecting the 5000 best corresponding features, SURF markedly improves the mosaic quality and sharply decreases run time compared with the gold‐standard ASIFT descriptor. Similarly, VMZ’s 2D image registration approach outperforms AutoStitch.

Historically, quadratic algorithms have been used where the images to be registered are unordered. We exploited the advantage of capturing video sequences, freely flown in one flight, so that each consecutive pair of frames has an overlapped region. This temporal continuity of the sequence allows us to use a linear algorithm. The imagery used here did not contain any significant changes in view, so we were able to generate a single mosaic from each sequence in linear time. However, it is important to account for situations where there are greater discontinuities in the frame sequence due to changes in the vehicle position relative to the field, camera view, or flight trajectory. This is especially true for freely or adaptively flown flights. This is where VMZ’s shot detection, or temporal segmentation, is important. Shot detection divides a whole sequence with discontinuities into a few meaningful and contiguous smaller sequences (the “shot” in VMZ). From each shot, one mini‐mosaic is generated.

Our results reflect the way the different feature descriptors operate. The ST descriptor performs a cruder match than other descriptors, because it finds edges and corners by relying on blocks of color gradients; thus, it consistently finds fewer features than either ASIFT or SURF and relies more heavily on RANSAC inliers (Appendix S4A, B). When the percentage of RANSAC inliers for the ST descriptor falls below 40%, VMZ‐Adaptive switches from the ST to the SURF descriptors; this occurs in almost half of the frames. Alternatively, ASIFT finds a large number of matching key points for affine transformations, but with a much greater cost. Among the descriptors used here, it runs more than 30 times more slowly and often does not produce the best mosaics, despite being regarded as the gold‐standard descriptor. Furthermore, ASIFT allows affine transformations to be matched, which negatively affects quality for repetitive patterns such as field crops. Although SURF extracts 14,000–15,000 features, VMZ‐SURF selects the 5000 strongest, resulting in more robust homographies with less noise in matching due to weaker key points. The VMZ pipeline uses RANSAC, which estimates the parameters of a model to distinguish outliers from inliers in a distribution of data, to filter key points; thus, each time RANSAC is run, different key points will be retained as inliers. Multiple samples of inliers using RANSAC might slightly improve selection of key points.

Another way of assessing the performance of the descriptors is to examine the scalar SSIM statistics (Table 1). The *SSIM_p_* score is a global measure of how well the entire mosaic is constructed. The *SSIM_p_* values for all the descriptors were similar, ranging from 0.98–0.99, with SURF consistently having the highest score. The *SSIM_f_* score computes the frame‐to‐frame similarity between the first frame and each successive frame at time *t* during mosaicking. Here, differences in the quality of mosaicking among the four descriptors are much more apparent, consistent with the localized errors observed in the mosaics. For two out of the three sequences, SURF again had the highest score and in the third was only marginally inferior to ASIFT. Thus, SURF is the most accurate descriptor for this imagery, as determined using several criteria.

Apart from repetitive plant features, several other factors can influence the quality of the mosaic produced by any of the methods compared here. The main optical factor is distortion due to the lens, particularly if it is asymmetric. We corrected the lens distortion prior to mosaicking (Appendix S3), which somewhat improved the mosaicking but consequently more starkly revealed the mechanical distortion. Mechanical distortion can occur if the orientation of the camera is not perpendicular to the zenith (in nadir view) or yawed relative to the nose of the aircraft (in oblique views). The empirical correction of gimbal distortion in the first frame of a video substantially improved the planarity of the resulting mosaic (data not shown) faster and at much less cost than using an SfM technique (Avola et al., 2018). Finally, many crop fields are not strictly planar, exhibiting multiple changes of elevation over their area. This is the major cause of the pinching and tilt of the right side of the mosaics in Appendix S3.

We demonstrate a faster, more robust algorithm for mosaicking aerial imagery of crop fields. After comparing the results from different descriptors, we conclude that VMZ‐SURF outperforms other descriptors for maize field imagery both in mosaic quality and run time. The robustness of this approach is achieved by exploiting the temporal consistency within the video sequence and utilizing a grouping technique to handle multiple coordinate systems for image registration. Currently, feature extraction and matching are responsible for around 88% of the total running time. Automatic correction for lens, gimbal, and elevation distortions, as well as the adaptation of a good blending method, might be helpful in reducing visual artifacts such as localized bending within the mosaic and illumination changes. In the future, we plan to extend our mosaicking algorithm to exploit parallelization, to make these corrections, and to support features from deep learning systems to further reduce the execution time and improve feature matching quality. This work is a first step in automatically measuring morphological and developmental phenotypes using freely or adaptively flown trajectories designed to directly capture that information, and to exploit the rapid computational times for the eventual dynamic scouting of production fields.

## AUTHOR CONTRIBUTIONS

R.A., K.P., F.B., and H.A. planned and designed the research for generating the mosaics. R.A. and K.P. designed and implemented the VMZ pipeline and mini‐mosaicking algorithm, and R.A. produced the results. D.E.K. collected the 2019 imagery, corrected it for lens distortion, and generated mosaics using only ASIFT for comparison with VMZ. A.E.S. contributed to the conceptualization of the work. T.K. designed and executed the maize experiments, collected the 2016 imagery, and contributed to conceptualization and algorithm formulation. All the authors contributed to the analysis of the results and writing the manuscript.

## Supporting information


**APPENDIX S1.** Mosaics of sequence DJI_0003.mov produced by AutoStitch (A) and VMZ’s three feature descriptors (B–D). A soybean field is located to the right of the maize field, with a pumpkin field located below it. (A) AutoStitch (3438 × 4032 pixels), (B) VMZ‐Adaptive (2074 × 2201 pixels), (C) VMZ‐ASIFT (2091 × 2198 pixels), (D) VMZ‐SURF (2083 × 2171 pixels). Sizes in parentheses are of the original high‐resolution mosaics; they are rescaled in the figure to make the field approximately the same size in each mosaic. Registration errors are visible in (A–C) as non‐square field geometries and distortion of the range/alley checkerboard. Poor color rendering due to the misregistration of the pixels prior to blending is seen as stripes of abrupt shifts in color over the ranges, particularly in (B), and paler pumpkins in the field below the maize field. Minor lens distortion is visible along the bottom of all four mosaics.

**APPENDIX S2.** Errors in field geometry and color blending in the DJI_0003.mov mosaics. The red box in (A) marks the lower edge of the field from the VMZ‐SURF mosaic that is scaled up in (B–E) for AutoStitch and VMZ’s three feature descriptors: AutoStitch (B), VMZ‐Adaptive (C), VMZ‐ASIFT (D), VMZ‐SURF (E). The left edge of the soybean field is actually parallel to the right edge of the maize field, as seen in panel (E). Poor color blending is visible in the bottom left corner of the soybeans.

**APPENDIX S3.** Mosaics of sequence DJI_00174.mov produced by AutoStitch (A) and VMZ’s different descriptors (B–D). The frames were corrected for lens distortion prior to mosaicking. (A) AutoStitch (8598 × 2182 pixels), (B) VMZ‐Adaptive (6496 × 2065 pixels), (C) VMZ‐ASIFT (7708 × 2074 pixels), (D) VMZ‐SURF (8796 × 2256 pixels). Sizes in parentheses are of the original high‐resolution mosaics; they are rescaled in the figure to make the field approximately the same size in each mosaic. Distortion of the field geometry arises from the registration errors previously noted and also from the field’s nonplanarity. The field is crowned near the center of the bottom edge. Uncorrected gimbal error contributes a bit to the distortion (data not shown).

**APPENDIX S4.** Distributions of feature statistics in sequences DJI_0026.mov and DJI_0003.mov. (A, B) Percentage of the number of features for the three descriptors used for the feature extraction, feature matching, and RANSAC filtration steps for DJI_0026.mov (A) and DJI_0003.mov (B). Here the *x*‐axes and *y*‐axes correspond to the different descriptors and the percentage of features after each step, respectively. (C, D) Number of features found by SURF after each processing step along the sequences in DJI_0026.mov (C) and DJI_0003.mov (D). Here the *x*‐axes and *y*‐axes correspond to the video frame number and the number of features, respectively.

**APPENDIX S5.** Further analysis of mosaic geometry and feature statistics in the Video Mosaicking and summariZation (VMZ) pipeline.

## Data Availability

The raw video files used in this work are available at CyVerse (https://datacommons.cyverse.org/browse/iplant/home/shared/) in the cornet_data folder. The direct URL for the data set was not available at the time of article publication

## APPENDIX 1 Algorithmic details of the Video Mosaicking and summariZation (VMZ) pipeline.

### Shot detection

Shot detection finds clusters of adjacent frames by looking for changes in the differences in the cumulative histogram of graph‐cut energy E(I). Two main clusters of frames are generated in this step. ${\mathrm{cluster}}_{0}$ groups contiguous frames that have no or very small changes in E(I) between two consecutive frames to form a shot, while ${\mathrm{cluster}}_{1}$ represents gradual or abrupt changes in scene (Eqn. 1):

(1) $$E\left(I\right)=\sum_{p}D_{c}\left(I_{p},L_{p}\right)+\sum_{p}\sum_{q}V_{\mathrm{pq}}\left(L_{p}\left(I_{p}\right),L_{q}\left(I_{q}\right)\right)$$

Here, $\sum_{p}D_{c}\left(I_{p},L_{p}\right)$ is the *data term* that sums the cost of assigning a cluster label $L_{p}$ to frame *I_p_*. The second term, $\sum_{p}\sum_{q}\sum_{\mathrm{pq}}\left(L_{p}\left(I_{p}\right),L_{q}\left(I_{q}\right)\right)$, is the *smoothness term* that computes the penalty of assigning frames $I_{p}$ and $I_{q}$ to cluster labels $L_{p}$ and $L_{q}$. More details of this algorithm can be found in Viguier et al. (2015).

### Feature descriptors

#### Structure tensor and normalized cross correlation

The 2D color structure tensor (ST) used here (Hafiane et al., 2008) can be defined in terms of the outer product of spatial gradients in each of the three RGB color channels of an image. The eigenvalues of the ST can be used as a local descriptor that provides useful information about the signal in orthogonal directions. This local descriptor finds the distinctive properties of an image such as edges and corners. As the magnitude of the ST response is directly related to the distinctiveness of an image, a prominent feature microblock is defined as a feature block that contains high responses. The feature microblocks are matched to a target image using normalized cross correlation (NCC), which matches a template or sub‐image to another image and returns the index of matched positions based on the similarity measurement between two images. NCC becomes useful when prominent feature blocks are found using the ST as a preprocessing step (Hafiane et al., 2008). The feature block from the source image is checked within a search window in the target image. The feature block is shifted by one pixel each time to cover all regions of the search window, and the NCC measure is used to find the best fit.

#### Scale‐invariant feature transform

The scale‐invariant feature transform (SIFT) (Lowe, 2004) is a local descriptor based on the appearance of an object at particular interest points. SIFT feature matching is done through a Euclidean distance–based nearest neighbor approach and a best‐bin‐first algorithm. In order to increase robustness in object matching, Hough transforms and least square minimizations are performed.

#### Speeded‐up robust features

The speeded‐up robust features (SURF) value is a local descriptor partly inspired by SIFT (Lowe, 2004; Bay et al., 2006). It uses a fast Hessian‐based blob detector to find distinctive key points. Once the points are identified, robust feature vectors are calculated based on the sum of the Haar wavelet response around the key points. These features are compared among two images to find similarities.

#### Affine scale‐invariant feature transform

Affine SIFT (ASIFT) (Morel and Yu, 2009) covers the four parameters from SIFT (scaling, orientation, illumination change, and affine distortion) and can also use the latitudinal and longitudinal angles of the orientation of the camera axis. ASIFT also introduced the notion of a transition tilt, which effectively measures the amount of distortion from one view to another using the camera axis parameters.

### Homography estimation

Given a scene that is approximately planar with one image I(x,y,k) and a later image I(u,v,t‐k), and given the spatial coordinates (x,y) in the first image, we need to calculate the new coordinates (u,v) in the second image with reference to the coordinate system of the first. Here, *t* corresponds to the time interval during which the images were taken, and *k* is a point in that temporal interval. The relationships between these coordinate systems are:

(2) $$\begin{array}{ccc} u & = & \frac{a_{1}x+a_{2}y+a_{0}}{c_{1}x+c_{2}y+1}\\ v & = & \frac{b_{1}x+b_{2}y+b_{0}}{c_{1}x+c_{2}y+1} \end{array}$$

The homography transformation matrix can be written as:

(3) $$\left[\begin{array}{c} u\\ v\\ w \end{array}\right]=\left[\begin{array}{ccc} a_{1} & a_{2} & a_{0}\\ b_{1} & b_{2} & b_{0}\\ c_{1} & c_{2} & 1 \end{array}\right]\left[\begin{array}{c} x\\ y\\ 1 \end{array}\right]$$

where *w* is a parameter. There are eight transformation parameters (*a*_1_, *a*_2_, *a*_0_, *b*_1_, *b*_2_, *b*_0_, *c*_1_, and *c*_2_) that are unknown but can be determined from four identical points shared by image I(x,y,k) and I(u,v,t‐k):

(4) $$\left[\begin{array}{cccccccc} x_{1} & y_{2} & 1 & 0 & 0 & 0 & ‐x_{1}u_{1} & ‐y_{1}u_{1}\\ x_{2} & y_{2} & 1 & 0 & 0 & 0 & ‐x_{2}u_{2} & ‐y_{2}u_{2}\\ x_{3} & y_{3} & 1 & 0 & 0 & 0 & ‐x_{3}u_{3} & ‐y_{3}u_{3}\\ x_{4} & y_{4} & 1 & 0 & 0 & 0 & ‐x_{4}u_{4} & ‐y_{4}u_{4}\\ 0 & 0 & 0 & x_{1} & y_{2} & 1 & ‐x_{1}v_{1} & ‐y_{1}v_{1}\\ 0 & 0 & 0 & x_{2} & y_{2} & 1 & ‐x_{2}v_{2} & ‐y_{2}v_{2}\\ 0 & 0 & 0 & x_{3} & y_{3} & 1 & ‐x_{3}v_{3} & ‐y_{3}v_{3}\\ 0 & 0 & 0 & x_{4} & y_{4} & 1 & ‐x_{4}v_{4} & ‐y_{4}v_{4} \end{array}\right]\left[\begin{array}{c} a_{1}\\ a_{2}\\ a_{0}\\ b_{1}\\ b_{2}\\ b_{0}\\ c_{1}\\ c_{2} \end{array}\right]=\left[\begin{array}{c} u_{1}\\ u_{2}\\ u_{3}\\ u_{4}\\ v_{1}\\ v_{2}\\ v_{3}\\ v_{4} \end{array}\right]$$

These eight unknowns can be found by solving Eqn. 4 using direct linear transformation (Abdel‐Aziz et al., 2015), provided that the corresponding points $\left(x_{1},y_{1}\right)$, $\left(x_{2},y_{2}\right)$, $\left(x_{3},y_{3}\right)$, and $\left(x_{4},y_{4}\right)$ from image I(x,y,k) and points $\left(u_{1},v_{1}\right)$, $\left(u_{2},v_{2}\right)$, $\left(u_{3},v_{3}\right)$, and $\left(u_{4},v_{4}\right)$ from image I(u,v,t‐k) are known.

### Quantitative evaluation of mosaicking performance

#### Structural similarity index

The structural similarity index measure (SSIM) provides a good measure of how well two registered images are aligned with one another, and has been used to evaluate mosaicking performance for crop growth monitoring (Bolaños and Edmeades, 1996). SSIM is defined in Eqn. 5:

(5) $$SSIM\left(I,J\right)=\frac{\left(2\mu_{I}\mu_{J}+C_{1}\right)\left(2\sigma_{I}\sigma_{J}+C_{2}\right)}{\left(\mu_{I}^{2}+\mu_{J}^{2}+C_{1}\right)\left(\sigma_{I}^{2}+\sigma_{J}^{2}+C_{2}\right)}$$

where for images *I* and *J*, $\mu_{I}$, $\mu_{J}$ are their local means, $\sigma_{I}$ and $\sigma_{J}$ their standard deviations, $\sigma_{I}\sigma_{J}$ their covariance, and $C_{1}$ and $C_{2}$ are constants. The SSIM scores range from 0 to 1, where the highest value corresponds to a perfect alignment in mosaicking or structural similarity, and the lowest value to badly warped images in the mosaic or very different structures.

To evaluate mosaicking performance, two different SSIM scores were calculated. First, *SSIM_f_* quantifies how well a warped frame at time *t* is aligned or structurally similar to an overlapping region of the initial mosaic computed at *t* = 1. In Eqn. 5, *I* and *J* become $M_{1}$ and $F_{t}^{\prime }$, where $M_{1}$ is the mosaic obtained from the base or first frame of the sequence and $F_{t}^{\prime }$ is the warped frame in base coordinates at time *t*. Higher values of *SSIM_f_* indicate that the warped frame is well aligned with the mosaic generated from the base frame. In contrast, *SSIM_p_* measures how well a warped frame at time *t* is aligned or structurally similar to the mosaic obtained from frames 1 to *t* − 1. In Eqn. 5, *I* and *J* become $M_{t‐1}$ and $F_{t}^{\prime }$, where $M_{t‐1}$ is the mosaic so far. Higher values of *SSIM_p_* indicate that the current frame $F_{t}^{\prime }$ is well aligned with the cumulative mosaic.

#### Scene integrity

Scene integrity is defined as the matching ratio between the output mosaic and the input scene (Eqn. 6) (Yang et al., 2015):

(6) $$\text{Scene integrity}=\frac{\text{no. of images in mosaic}}{\text{no. of images in scene}}$$

Scene integrity ranges from 0 to 1, where 0 denotes complete failure (no two images can be matched and registered) and 1 indicates all images are used in matching, registration, warping, and mosaicking. Scene integrity indicates good performance in image matching and the algorithm’s effectiveness in using the frames.

### The VMZ algorithm

#### Terminology

We define the following terms.

- *Base*: The first frame of a shot is called the *base* frame and its coordinates will be used as the mini‐mosaic coordinates (Fig. 3D, E).
- *Group*: A shot is divided into several groups based on the number of frames and the motion and displacement among the frames (Fig. 3C). A new group is formed if the displacement between the reference frame and the current frame is greater than the threshold (motion among frames and number of frames in a group), or if the number of frames in the group is greater than 40.
- *Reference*: The first frame of a group is called the *reference* frame (Fig. 3D, E).
- *Current‐to‐reference homography* (*H_CR*): The homography matrix that aligns any *Frame_i_* in a group to the reference frame of the group (Fig. 3D) and is calculated directly between *Frame_i_* and its reference, *Frame_j_*.
- *Reference‐to‐reference homography* (*H_RR*): The homography matrix that aligns any reference frame of a group to the reference frame of the previous group (Fig. 3E). It is calculated directly between two consecutive reference frames, *Frame_j_* and *Frame_k_*, the first frames of groups *m* and *m* − 1, respectively.
- *Reference‐to‐base homography* (*H_RB*): The homography matrix that aligns any reference frame to the *base* or first frame of a shot. This is obtained by multiplying the sequence of *H_RR*s over the shot, as shown in Eqn. 7:
  (7) $$\begin{array}{ccc} H\_RB_{1\leftarrow j} & = & H\_RR_{1\leftarrow 1}*H\_RR_{1\leftarrow 2}*H\_RR_{2\leftarrow 3}*...*H\_RR_{j‐2\leftarrow j‐1}*H\_RR_{j‐1\leftarrow j}\\ & = & H\_RB_{1\leftarrow j‐1}*H\_RR_{j‐1\leftarrow j}. \end{array}$$
- *Current‐to‐base homography* (*H_CB*): The homography matrix that aligns any *Frame_i_* in a group to the base frame of the shot. This matrix is required for transforming any frame to the base coordinate system (Eqn. 8):
  (8) $$\begin{array}{ccc} H\_CB_{1\leftarrow i} & = & H\_CR_{j\leftarrow i}*H\_RB_{1\leftarrow j}. \end{array}$$

### ALGORITHM 1. MINI‐MOSAIC GENERATION

1. j← 1
2. no_img_in_group← 0
3. $Reference_{j}$ ← $Frame_{1}$
4. $H\_RR_{1\leftarrow 1}\leftarrow$ [1 0 0; 0 1 0; 0 0 1]
5. $H_{RB_{1\leftarrow 1}}\leftarrow$ $H_{RR_{1\leftarrow 1}}$
6. fori← 1 *to n* do
7. $[H\_CR_{j\leftarrow i},displacement]\leftarrow$ $getHomography(Reference_{j},Frame_{i})$
8. $if\left(no\_img\_in\_group>40\right)or\left(displacement>dispThresh\right)then$
9. $H\_RR_{j\leftarrow j+1}\leftarrow$ $getHomography(Reference_{j},Frame_{i})$
10. j← j+1
11. $H\_RB_{1\leftarrow j}\leftarrow$ $H\_RB_{1\leftarrow j‐1}*H\_RR_{j‐1\leftarrow j}$
12. $Reference_{j}\leftarrow Frame_{i}$
13. no_img_in_group←0
14. else
15. $H\_CB_{1\leftarrow i}\leftarrow$ $H\_CR_{i\leftarrow j}*H\_RB_{1\leftarrow j}$
16. i← i+1
17. no_img_in_group ←no_img_in_group+1
18. Corners←estimateCorners(H_CB)
19. MM←calculateCanvas(Corners)
20. fori← 1 *to n* do
21. $WFrame_{i}\leftarrow warpImage\left(Frame_{i},H\_CB_{1\leftarrow j}\right)$
22. $MM\_ROI\leftarrow getROIfromCanvas(MM,Corners_{i})$
23. $MM\_ROI\leftarrow blend(MM\_ROI,WFrame_{i})$
24. MM←updateMinimosaic(MM,MM_ROI)
