# Corrigendum

**DOI:** 10.1002/aps3.11397

**Published:** 2020-10-30

In our paper, “Robust mosaicking of maize fields from aerial imagery,” the funding information was incorrect. A previous grant (Air Force Research Laboratory grant no. FA8750‐14‐2‐0072) was listed instead of the current grants (Army Research Laboratory Cooperative Agreement W911NF1820285 and Army Research Office DURIP W911NF1910181) and fellowship support was not included for one of the authors.

The first sentence of the Acknowledgments has been corrected as:

The authors gratefully acknowledge partial support from the U.S. Army Research Laboratory (cooperative agreement W911NF1820285), the Army Research Office (DURIP W911NF1910181), an Executive Women’s Forum doctoral fellowship through the University of Missouri College of Engineering (to R.A.), and the National Corn Growers’ Association.

We apologize for the error.
