# Supplementary material for: Robust mosaicking of maize fields from aerial imagery
Source: Appl Plant Sci. 2020 Sep 10;8(8):e11387. doi: 10.1002/aps3.11387 (PMC7507512; doi:10.1002/aps3.11387)

DOI 10.1002/aps3.11387

APPENDIX S1. Mosaics of sequence DJI_0003.mov produced by AutoStitch (A) and VMZ's three feature descriptors (B–D). A soybean field is located to the right of the maize field, with a pumpkin field located below it. (A) AutoStitch (3438 × 4032 pixels), (B) VMZ-Adaptive (2074 × 2201 pixels), (C) VMZ-ASIFT (2091 × 2198 pixels), (D) VMZ-SURF (2083 × 2171 pixels). Sizes in parentheses are of the original high-resolution mosaics; they are rescaled in the figure to make the field approximately the same size in each mosaic. Registration errors are visible in (A–C) as non-square field geometries and distortion of the range/alley checkerboard. Poor color rendering due to the misregistration of the pixels prior to blending is seen as stripes of abrupt shifts in color over the ranges, particularly in (B), and paler pumpkins in the field below the maize field. Minor lens distortion is visible along the bottom of all four mosaics.

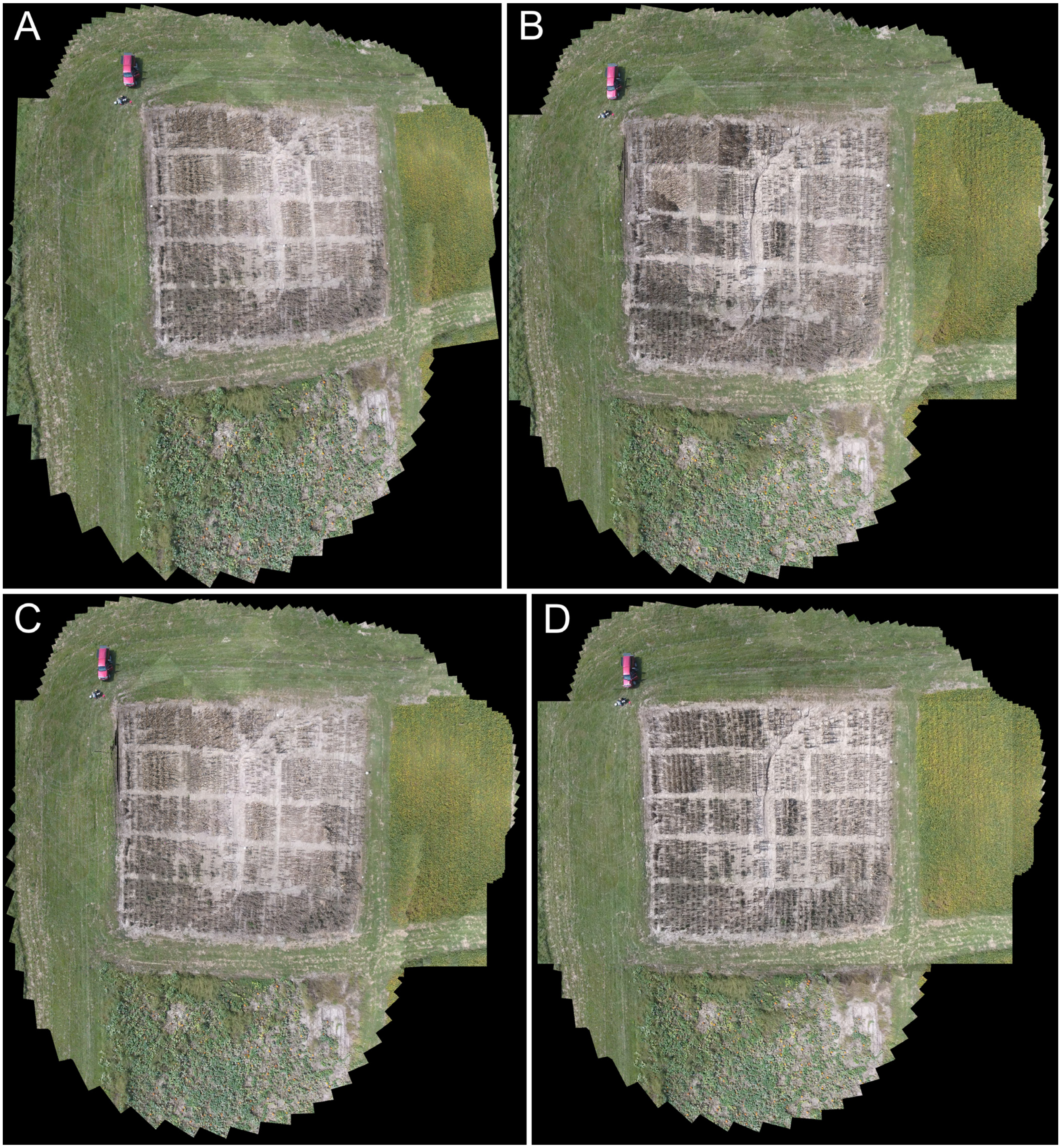

Supplement: Supplementary file 1 — APPENDIX S1. Mosaics of sequence DJI_0003.mov produced by AutoStitch (A) and VMZ’s three feature descriptors (B–D). A soybean field is located to the right of the maize field, with a pumpkin field located below it. (A) AutoStitch (3438 × 4032 pixels), (B) VMZ‐Adaptive (2074 × 2201 pixels), (C) VMZ‐ASIFT (2091 × 2198 pixels), (D) VMZ‐SURF (2083 × 2171 pixels). Sizes in parentheses are of the original high‐resolution mosaics; they are rescaled in the figure to make the field approximately the same size in each mosaic. Registration errors are visible in (A–C) as non‐square field geometries and distortion of the range/alley checkerboard. Poor color rendering due to the misregistration of the pixels prior to blending is seen as stripes of abrupt shifts in color over the ranges, particularly in (B), and paler pumpkins in the field below the maize field. Minor lens distortion is visible along the bottom of all four mosaics. [file APS3-8-e11387-s001.pdf]
