# Supplementary material for: Robust mosaicking of maize fields from aerial imagery
Source: Appl Plant Sci. 2020 Sep 10;8(8):e11387. doi: 10.1002/aps3.11387 (PMC7507512; doi:10.1002/aps3.11387)

DOI 10.1002/aps3.11387

APPENDIX S2. Errors in field geometry and color blending in the DJI_0003.mov mosaics. The red box in (A) marks the lower edge of the field from the VMZ-SURF mosaic that is scaled up in (B–E) for AutoStitch and VMZ's three feature descriptors: AutoStitch (B), VMZ-Adaptive (C), VMZ-ASIFT (D), VMZ-SURF (E). The left edge of the soybean field is actually parallel to the right edge of the maize field, as seen in panel (E). Poor color blending is visible in the bottom left corner of the soybeans.

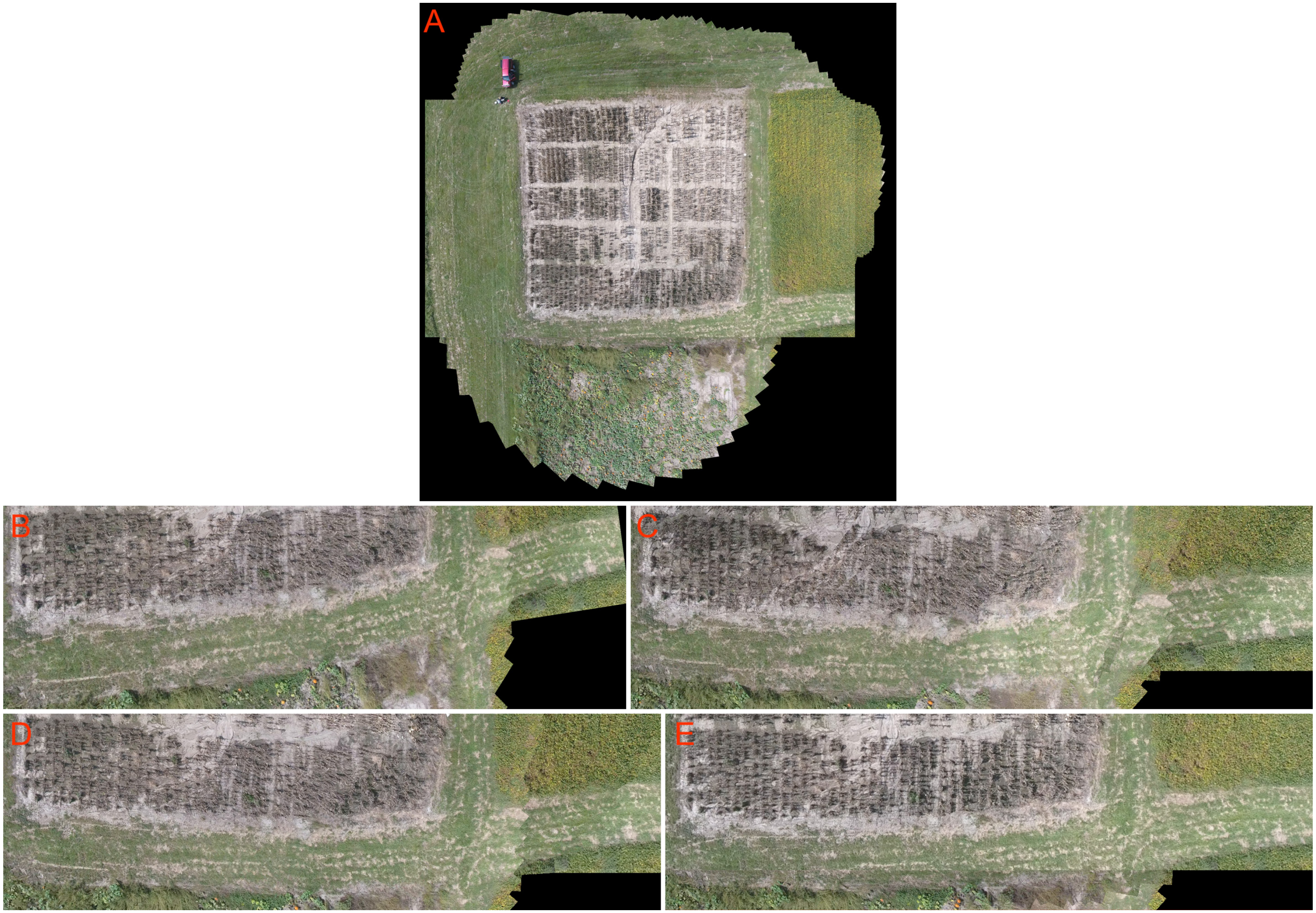

Supplement: Supplementary file 2 — APPENDIX S2. Errors in field geometry and color blending in the DJI_0003.mov mosaics. The red box in (A) marks the lower edge of the field from the VMZ‐SURF mosaic that is scaled up in (B–E) for AutoStitch and VMZ’s three feature descriptors: AutoStitch (B), VMZ‐Adaptive (C), VMZ‐ASIFT (D), VMZ‐SURF (E). The left edge of the soybean field is actually parallel to the right edge of the maize field, as seen in panel (E). Poor color blending is visible in the bottom left corner of the soybeans. [file APS3-8-e11387-s002.pdf]
