# Supplementary material for: Robust mosaicking of maize fields from aerial imagery
Source: Appl Plant Sci. 2020 Sep 10;8(8):e11387. doi: 10.1002/aps3.11387 (PMC7507512; doi:10.1002/aps3.11387)

Aktar et al.—Applications in Plant Sciences 2020 8(8)—Data Supplement S3. Page 1 of 1.
DOI 10.1002/aps3.11387

APPENDIX S3. Mosaics of sequence DJI_00174.mov produced by AutoStitch (A) and VMZ's different descriptors (B–D). The frames were corrected for lens distortion prior to mosaicking. (A) AutoStitch (8598 × 2182 pixels), (B) VMZ-Adaptive (6496 × 2065 pixels), (C) VMZ-ASIFT (7708 × 2074 pixels), (D) VMZ-SURF (8796 × 2256 pixels). Sizes in parentheses are of the original high-resolution mosaics; they are rescaled in the figure to make the field approximately the same size in each mosaic. Distortion of the field geometry arises from the registration errors previously noted and also from the field's nonplanarity. The field is crowned near the center of the bottom edge. Uncorrected gimbal error contributes a bit to the distortion (data not shown).

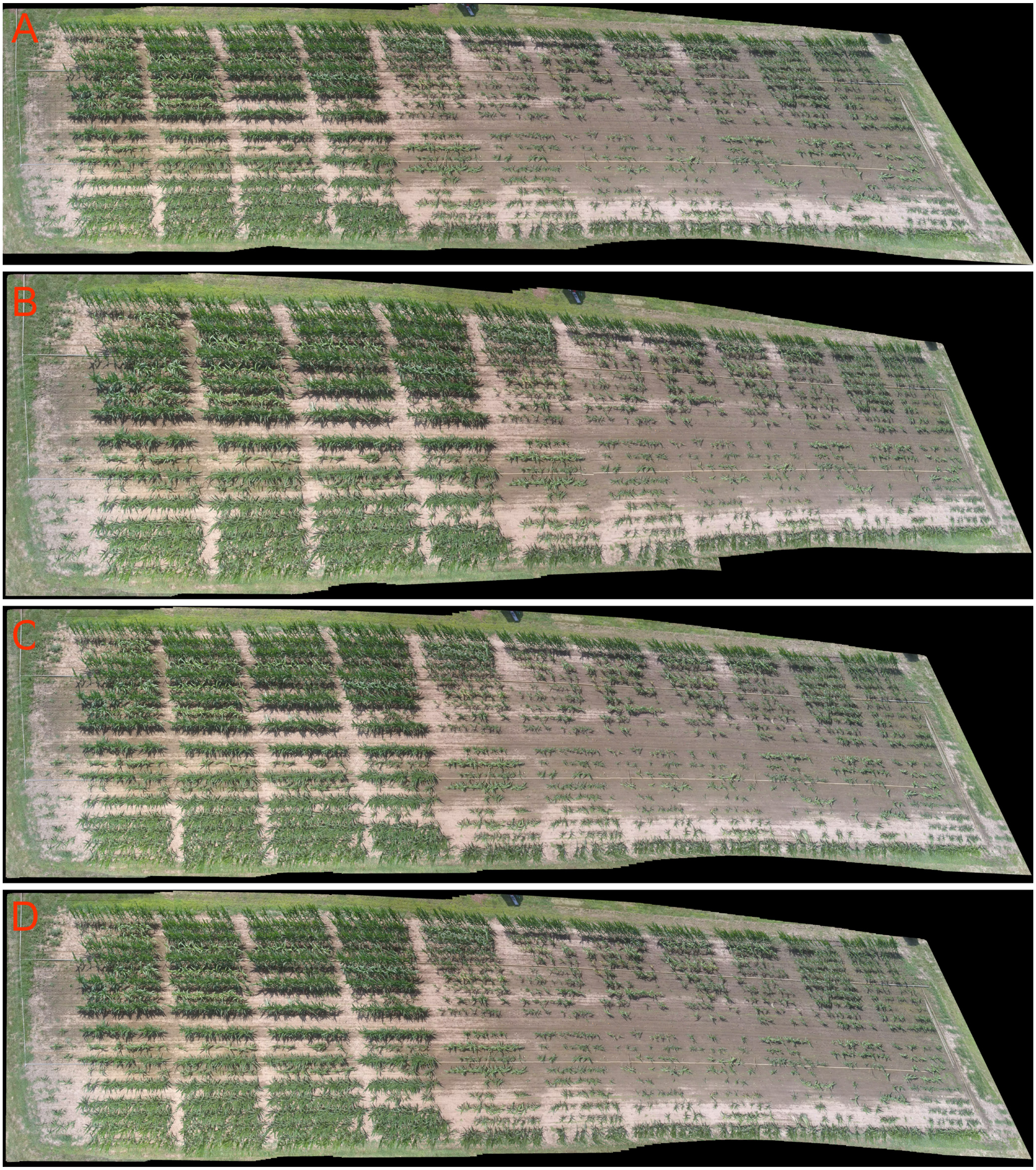

Supplement: Supplementary file 3 — APPENDIX S3. Mosaics of sequence DJI_00174.mov produced by AutoStitch (A) and VMZ’s different descriptors (B–D). The frames were corrected for lens distortion prior to mosaicking. (A) AutoStitch (8598 × 2182 pixels), (B) VMZ‐Adaptive (6496 × 2065 pixels), (C) VMZ‐ASIFT (7708 × 2074 pixels), (D) VMZ‐SURF (8796 × 2256 pixels). Sizes in parentheses are of the original high‐resolution mosaics; they are rescaled in the figure to make the field approximately the same size in each mosaic. Distortion of the field geometry arises from the registration errors previously noted and also from the field’s nonplanarity. The field is crowned near the center of the bottom edge. Uncorrected gimbal error contributes a bit to the distortion (data not shown). [file APS3-8-e11387-s003.pdf]
