# Supplementary material for: Robust mosaicking of maize fields from aerial imagery
Source: Appl Plant Sci. 2020 Sep 10;8(8):e11387. doi: 10.1002/aps3.11387 (PMC7507512; doi:10.1002/aps3.11387)

DOI 10.1002/aps3.11387

APPENDIX S4. Distributions of feature statistics in sequences DJI_0026.mov and DJI_0003.mov. (A, B) Percentage of the number of features for the three descriptors used for the feature extraction, feature matching, and RANSAC filtration steps for DJI_0026.mov (A) and DJI_0003.mov (B). Here the *x*-axes and *y*-axes correspond to the different descriptors and the percentage of features after each step, respectively. (C, D) Number of features found by SURF after each processing step along the sequences in DJI_0026.mov (C) and DJI_0003.mov (D). Here the *x*-axes and *y*-axes correspond to the video frame number and the number of features, respectively.

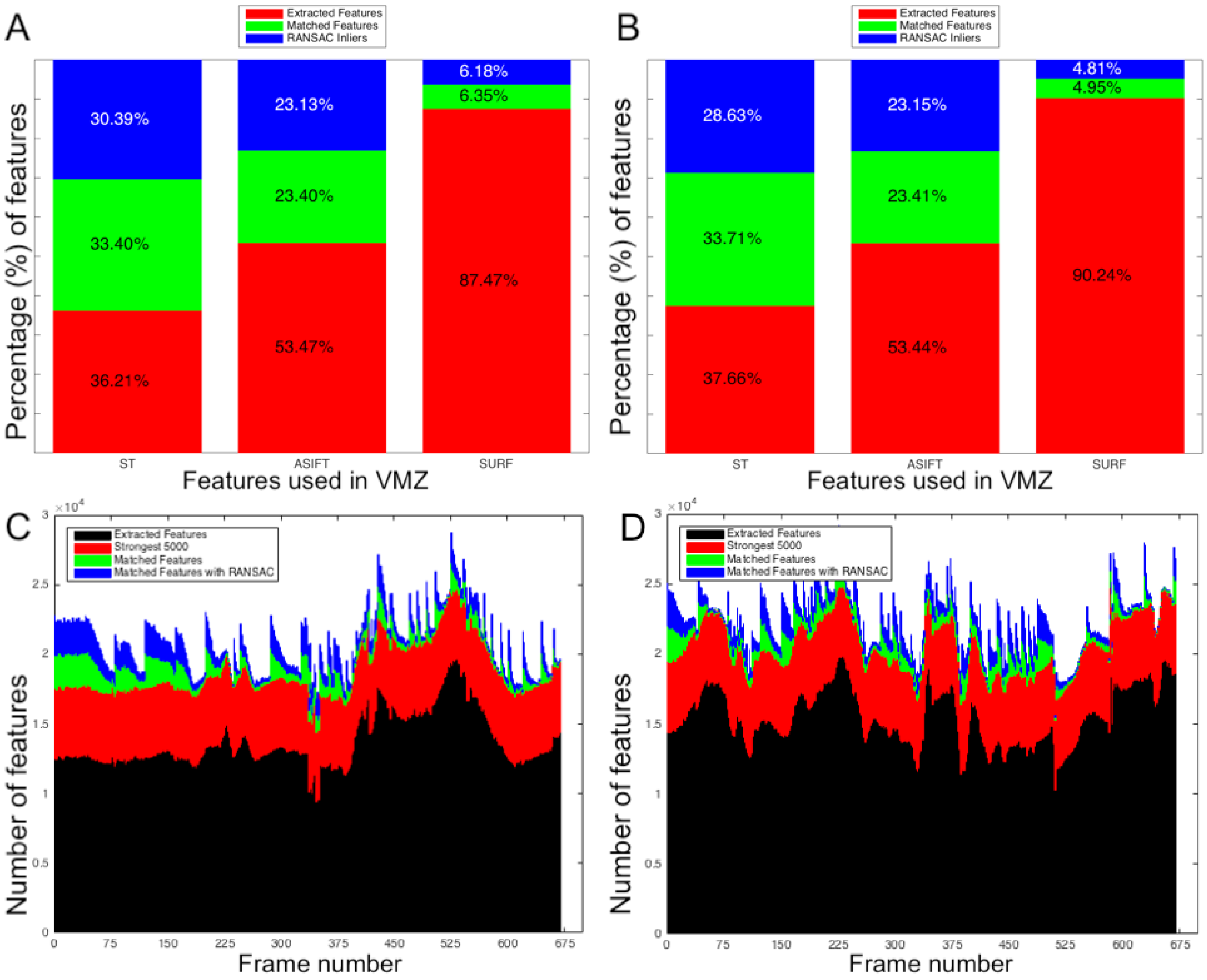

Supplement: Supplementary file 4 — APPENDIX S4. Distributions of feature statistics in sequences DJI_0026.mov and DJI_0003.mov. (A, B) Percentage of the number of features for the three descriptors used for the feature extraction, feature matching, and RANSAC filtration steps for DJI_0026.mov (A) and DJI_0003.mov (B). Here the x‐axes and y‐axes correspond to the different descriptors and the percentage of features after each step, respectively. (C, D) Number of features found by SURF after each processing step along the sequences in DJI_0026.mov (C) and DJI_0003.mov (D). Here the x‐axes and y‐axes correspond to the video frame number and the number of features, respectively. [file APS3-8-e11387-s004.pdf]
