# Supplementary material for: Robust mosaicking of maize fields from aerial imagery
Source: Appl Plant Sci. 2020 Sep 10;8(8):e11387. doi: 10.1002/aps3.11387 (PMC7507512; doi:10.1002/aps3.11387)
Supplement: Supplementary file 5 — APPENDIX S5. Further analysis of mosaic geometry and feature statistics in the Video Mosaicking and summariZation (VMZ) pipeline. [file APS3-8-e11387-s005.docx]

**APPENDIX S5.** Further analysis of mosaic geometry and feature statistics in the Video Mosaicking and summariZation (VMZ) pipeline.

**Distributions of feature statistics**

We examined the total distributions of the features found by each descriptor to determine if different video sequences have different optimal feature extraction methods (Appendix S4A, B). Each stacked bar represents the number of extracted features, matched features, and selected random sample consensus (RANSAC) inliers found by the descriptor. The distributions for each descriptor are consistent in both sequences. Structure tensor (ST), which matches each key point in the reference frame to the other frame, finds about an equal number of the three types. The affine scale-invariant feature transform (ASIFT) and speeded up robust features (SURF) descriptors find features in each of the two frames, and then match those. As a result, compared to ST, the proportion of extracted features increases. SURF is more efficient than ASIFT, leaving fewer features that must be found by matching and RANSAC elimination. This accelerates VMZ without negatively affecting homography estimation. Appendix S4C, D present the features found by SURF at each sampled frame of the videos. Consistent with the overall distributions of features, SURF’s selection of the 5000 strongest features throughout the video sequences reduces the work feature matching and RANSAC elimination must do.

**Errors in field geometry and color blending in DJI_0003.mov**

Appendix S1 shows the mosaicking errors that occurred in DJI_0026.mov are also found in DJI_ 0003.mov. Appendix S1A–C illustrate foreshortening of the fields’ geometry by AutoStitch, VMZ-Adaptive, and VMZ-ASIFT. In reality, the maize field is essentially square, with its right edge parallel to the left edge of the soybean field and a regular row/alley checkerboard. VMZ-Adaptive and VMZ-ASIFT recover the fields’ perimeters, internal geometry, and checkerboard more accurately than AutoStitch, but not as well as VMZ-SURF. Similar foreshortening of the top and right edges of the pumpkin field below the maize field are also visible. None of the three fields’ mosaics computed by AutoStitch, VMZ-Adaptive, and VMZ-ASIFT are rescued by the spontaneous fiducials of the ripe pumpkins at the bottom.

**Color blending errors signal poor registration during mosaicking**

The bottom of the maize field and its neighbors in the VMZ-SURF mosaic shown in Appendix S2A is enlarged in Appendix S2B–E. VMZ-Adaptive produces multiple dark regions in the maize field, and in the soybean field, it produces the largest patch of lighter color with washed out plants (Appendix S2C). VMZ-ASIFT has fewer and lighter dark patches in the maize field and a smaller region of washout in the soybean field (Appendix S2D). AutoStitch falls in between, suggesting its greater distortion is compensated for by a superior blending algorithm (Appendix S2B). VMZ-SURF’s dark patches geometrically align with the maize rows, consistent with the genetic varieties planted there, and the soybean field exhibits the least fading (Appendix S2E). The pumpkin field below the maize field follows these trends, judging by pumpkin color and sharpness.

**Geometric errors after correction for lens distortion in DJI_0174.mov**

Appendix S3 uses a video corrected for lens distortion prior to mosaicking. The rectangular field is mosaicked as trapezoidal by all four algorithms. VMZ-Adaptive introduces the most distortion and AutoStitch the least, twisting the right edge of the field less than VMZ with any descriptor. VMZ-ASIFT and VMZ-SURF distort the field geometry about equally. Consistent with the trapezoidal distortion, the plants along the upper edge of the field appear at acute angles to the ground in all four mosaics. All four algorithms preserve the row/alley checkerboard about equally well.
